# Genomics and adaptation in forest ecosystems

**DOI:** 10.1007/s11295-022-01542-1

**Published:** 2022-02-09

**Authors:** Charalambos Neophytou, Katrin Heer, Pascal Milesi, Martina Peter, Tanja Pyhäjärvi, Marjana Westergren, Christian Rellstab, Felix Gugerli

**Affiliations:** 1 Institute of Silviculture, Department of Forest and Soil Sciences, University of Natural Resources and Life Sciences (BOKU), Peter-Jordan-Str. 82, A-1190, Vienna, Austria; 2 Albert-Ludwigs Universität Freiburg, Forest Genetics, Bertoldstraße 17, D-79098 Freiburg, Germany; 3 Department of Ecology and Genetics, Evolutionary Biology Centre, Uppsala University, Norbyvägen 18D, SE 752 36 and ScilifeLab, Uppsala, Sweden; 4 Swiss Federal Research Institute WSL, Zürcherstrasse 111, CH–8903 Birmensdorf, Switzerland; 5 Department of Forest Sciences, University of Helsinki, Latokartanonkaari 7, FI-00014 Helsinki, Finland; 6 Slovenian Forestry Institute, Večna pot 2, SI-1000 Ljubljana, Slovenia

**Keywords:** Gene conservation, Genetic diversity, Local adaptation, Pathogens, Selection, Mycorrhizal fungi

## Abstract

Rapid human-induced environmental changes like climate warming represent a challenge for forest ecosystems. Due to their biological complexity and the long generation time of their keystone tree species, genetic adaptation in these ecosystems might not be fast enough to keep track with conditions changing at such a fast pace. The study of adaptation to environmental change and its genetic mechanisms is therefore key for ensuring a sustainable support and management of forests. The 4-day conference of the European Research Group EvolTree (https://www.evoltree.eu) on the topic of “Genomics and Adaptation in Forest Ecosystems” brought together over 130 scientists to present and discuss the latest developments and findings in forest evolutionary research. Genomic studies in forest trees have long been hampered by the lack of high-quality genomics resources and affordable genotyping methods. This has dramatically changed in the last few years; the conference impressively showed how such tools are now being applied to study past demography, adaptation and interactions with associated organisms. Moreover, genomic studies are now finally also entering the world of conservation and forest management, for example by measuring the value or cost of interspecific hybridization and introgression, assessing the vulnerability of species and populations to future change, or accurately delineating evolutionary significant units. The newly launched conference series of EvolTree will hopefully play a key role in the exchange and synthesis of such important investigations.

**Supplementary Information:**

The online version contains supplementary material available at 10.1007/s11295-022-01542-1.

## Introduction

Forests represent key ecosystems world-wide. They cover an estimated 30% of the Earth’s land surface, they harbour tremendous biodiversity at all levels (genes, species, ecosystems, and ecosystem services) and play a prominent role in the global water and chemical cycles, notably carbon. Not the least, forests are economically important for their wood and non-wood products, for protection of infrastructure and for recreation, and as such they fulfil manifold functions for human well-being.

**Fig. 1:**
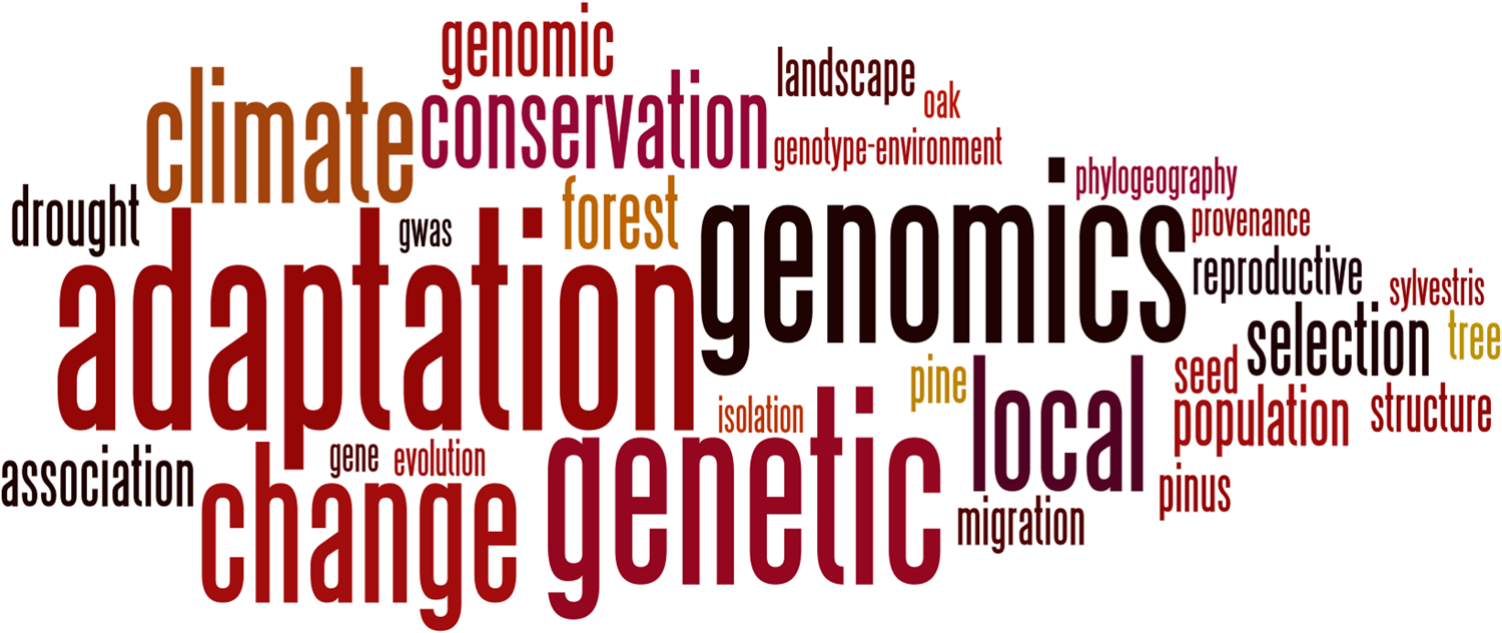
Word cloud of the keywords provided for all oral and poster presentations. Created with Wordle (https://mrfeinberg.com).

In recent years, genomic research has made tremendous progress in studying patterns and processes to elucidate aspects of evolutionary biology at several spatial and temporal scales. Among many others, the European Research Group EvolTree (https://www.evoltree.eu) has widely contributed to various aspects of forest genomic research. This self-supporting network of research institutes across mostly European countries has recently launched a new conference series to address a broad palette of topics in the context of genetic and genomic research in forest ecosystems, be it at the level of single species, communities or trophic interactions. The inaugural conference of this series was held at the Swiss Federal Research Institute WSL, Birmensdorf, in mid-September 2021, entitled “Genomics and Adaptation in Forest Ecosystems”. Under the restrictions given by the COVID-19 regulations, the conference organizers were challenged by establishing a hybrid event, allowing both on-site and online participation. This format enabled scientific exchange and personal contacts even across continents and the opportunities for interaction given by this conference were well received by the 132 participants from 27 nations. Over 4 days, the six topical sessions with 34 oral presentations and 23 posters (Table S1) covered complementary facets (Figure 1) of how individuals, populations and species cope with their environment. A wide range of approaches highlighted how diverse not only forests and their organisms are, but also the methodological toolboxes applied in forest genomics research. The latter was exemplified by two complementary teaching lectures on the availability and practical use of environmental data by Benjamin Dauphin (WSL, Switzerland) and on the population genetic simulation tools Nemo and Nemo-age by Fred Guillaume (University of Helsinki, Finland; Guillaume and Rougement 2006, Cotto et al. 2020). The conference also included an excursion for on-site participants to the Forest Lab Zürich (https://www.waldlabor.ch/).

In the subsequent sections, we recall the main findings of the work that was presented during the conference, either in one of the oral sessions or during the extended poster presentations. Detailed information on the individual contributions may be found in the online book of abstracts (Rellstab et al. 2021).

## Climate change genomics

Forests are particularly affected by rapid climatic change because of the longevity and the often limited seed dispersal of their keystone tree species. In the last decade, the development of next-generation sequencing technologies and of new analytical frameworks has opened a wealth of new perspectives in climate change genomics.

John Kelly (keynote, University of Kansas, USA) opened the session by presenting a new approach to predict allele frequency change in natural populations using a model incorporating fitness-effect-estimates from millions of markers in the short-lived model plant *Mimulus guttatus*. Although quantifying fitness in forest trees can be challenging, adaptive loci can still be identified by studying how allele frequencies change along environmental gradients through genotype–environment association (GEA) studies or are associated to adaptive phenotypic variations using genome-wide association studies (GWAS). An example of the former was provided by Jill Sekely (Philipps-University, Marburg, Germany) that identified the main climatic drivers of adaptation of *Nothofagus pumilio* along a 2000-km latitudinal transect in the Andean. Domitille Coq--Etchegaray (INRAE, France) used a GWAS on metabolomics data to evidence that micro-characters, such as leaf specialized metabolites that are notably involved in response to abiotic stress, can have an oligogenic architecture in sessile oak (*Quercus petraea*). Gaining insight into the relationships between genotypes, phenotypes and environments can help refine predictive models of species distribution under various climate change scenarios. Brandon Lind (University of British Columbia, Canada) compared the performances of various models of genetic offset by confronting their predictions with actual data from extensive common gardens of jack pine (*Pinus banksiana*) and Douglas fir (*Pseudotsuga menziesii*).

Species share not only environment but also history and a still open question is the extent of convergent adaptation. Pooja Singh (University of Calgary, Canada) investigated such a question by comparing the genomic signatures of adaptation to the same climate variables in eight coniferous species from two continents. Genomic studies have also evidenced the extent of hybridization between sister species or ecotypes that can also allow for the introgression of adaptive alleles as shown by Rakefet David-Schwartz (Volcani Center, Israel): Hybrids between *Pinus brutia* and *Pinus halepensis* resist drought better than their parental species.

To conclude, elucidating the relationships between the genomic level and higher levels of organization with the environment is of paramount importance to understand, or even predict and anticipate the impact of climate change on forest trees. Practical outcomes of climate change genomics notably include the development of genomically informed conservation and breeding strategies.

## Genomics of interactions

Organisms do not live and evolve in isolation, but interact with various living beings of their own and other kinds throughout their life histories. Long-lived trees as the foundation species of forest ecosystems provide a matrix of resources and habitats for associated organisms, with interactions ranging from beneficial to detrimental. The session “Genomics of interactions” addressed these complex interplays at the intraspecific and inter-kingdom levels and at diverse spatial and temporal scales. At the intraspecific level, Matthieu Tiret (Uppsala University, Sweden) discussed implementing group selection in tree breeding programs, because intraspecific competition of neighbouring trees proved to affect stand performance. At the interkingdom level, focusing on the mycorrhizal symbiosis, the keynote of Annegret Kohler (INRAE, France) highlighted how symbiotic lifestyle and interaction with host plants have shaped fungal genomes and transcriptomes. Besides a reduction in plant cell wall-degrading genes as a consequence of symbiosis, a heterogenous set of conserved genes that predate mycorrhizal evolution as well as new, lineage-specific genes were independently adopted for symbiosis function in diverse fungal lineages. This talk also showed that genes expressed in symbiosis setup seem not to point towards a mutualistic relationship, but rather resemble pathogenic interactions. For example, effector-like proteins are secreted also by mutualists and interfere with signalling pathways to reduce the hosts’ defence reactions. An elegant approach of finding the genetic basis of ectomycorrhizal traits was presented by Benjamin Dauphin (WSL, Switzerland). Progenies of a single fungal fruitbody interacting with a poplar clone revealed wide variation in phenotypic traits such as the proportion of roots colonized, ranging from incompatible to fully compatible strains. Transcriptome data from symbiotic tissues supported these strain-specific differences, and GWAS analyses are underway to detect responsible gene variants. Similarly, Isacco Beritognolo (CNR, Italy) presented an extreme-phenotype GWAS identifying genetic determinants that underlie the resistance of sweet chestnut to one of its most damaging pests, the Asian chestnut gall wasp. Such studies may assist in the genetic improvement of chestnut and development of effective methods to control this parasite. Renate Heinzelmann (University of British Columbia, Canada) finally made clear that not only genetic variation in host trees and associated organisms, but also the environment determines the outcome of plant–pathogen interactions. *Dothistroma* needle blight in North American pine forests indicates spatial patterns of genotype–genotype–phenotype interactions, the knowledge of which might help predicting disease outbreaks in future climate as well as managing forests using assisted migration.

## Past demography and post-glacial recolonization

This session addressed the question of how past geological and ecological changes have affected the genetic diversity of forest trees at the molecular level. The answer, as highlighted by the presentations, is that the effect of past changes in habitat and distribution will depend on the spatial, temporal and phylogenetic scale. The presentations covered time scales from millions of years to just a few generations, from continental to local scale, and from tropical to boreal regions.

Genome-wide methods are currently used in a large variety of tree species, such as exome capture of hundreds of genes across a whole subgeneric clade, across a large part of coding genes (e.g. Jade Bruxaux, Umeå University, Sweden) or even the whole genome (Desanka Lazic, Georg-August University of Göttingen, Germany). Moving from organellar markers to large amounts of nuclear loci has also improved the spatial and temporal resolution of demographic analyses of forest tree species.

In the long-term and at the continental scale, the effect of habitat shrinkage can positively affect diversity: global reduction in tropical rainforest area during the past millions of years has increased diversification of the tree species within the *Berlinia* clade (Fabaceae) and resulted in several independent shifts from forest to savanna habitat (Dario Ojeda, NIBIO, Norway). At the species level, there are indications of past population growth, e.g. in the widely distributed *Pinus sylvestris*, during the past million years and after the last glacial maximum (Chedly Kastally, University of Helsinki, Finland). On the other hand, in species with a more restricted distribution and niche, like *Pinus cembra*, the current speed of change seems more rapid than the capacity of genomic change, and there is a risk of non-adaptedness over a short time scale (Felix Gugerli, WSL, Switzerland; Dauphin et al. 2021).

Finally, an important message from the keynote speaker Parul Johri (Arizona State University, USA) was that especially in compact genomes, inference of demography alone, without accounting for negative background selection in linked sites, easily results in mis-inference of population growth. The proposed solution is the joint inference of both demographic and selection parameters, e.g. using approximate Bayesian computation. In many tree species, this may be challenging because fragmented reference genomes limit the acquisition of informative linkage disequilibrium estimates and other statistics on physical proximity of different loci and genes. However, as genome assemblies get better with long-read sequencing techniques, this may be a passing problem.

## Innovative methods and approaches

Studying the genomic basis of adaptation and understanding the dynamics of adaptation in times of climate change poses many challenges to forest geneticists due to the high complexity at the phenotypic and genomic level in many tree species. In this session, novel approaches for some of these challenges were presented. The keynote speaker Katie Lotterhos (Northeastern University, USA) discussed a new methodology to address covariance between genotype and environment, which is a result of the joint variability between genotypic and environmental effects on phenotypes. Moreover, she highlighted how genetic architecture and demography can result in the evolution of phenotypic clines without clines in causal allele frequencies. This suggests that commonly used methods to study GEA may be limited in their ability to discover the genetic architecture of adaptation.

Piyal Karunarathne (Uppsala University, Sweden) presented a statistical approach to detect and quantify copy number variations (CNVs), building on the HDplot approach previously introduced by McKinney et al. (2017). He implemented their approach in an easy-to-use R package (https://github.com/piyalkarum/rCNV) that allows running the whole workflow from the .vcf file to the detection of duplicates.

Sandra Cervantes (University of Oulu, Finland) discussed the possibility that selection might affect genes differently when they are predominantly expressed in haploid vs. diploid tissues. For her study, she focused on the haploid conifer megagametophyte. While here results indicated that purifying selection is more effective in haploid tissue, she could not detect the expected reduced diversity of genes predominantly expressed in the megagametophyte (Cervantes et al. 2021).

Using genomic data to predict a tree phenotype is a vital research field with high relevance for tree breeding. Abdou Rahmane Wade (INRAE, France) presented a new approach that integrates genomic and gene expression data to predict tree phenotypes in *Populus nigra*. He highlighted the importance of eQTLs (genomic variants predicting gene expression) as potential candidates for redundancy in such integrative approaches that could negatively impact prediction accuracy.

While multi-generation experiments are hardly feasible for most forest trees, the question of when and how selection acts in the tree’s life cycle remains an important question and challenging research task. Juan Jose Robledo-Arnuncio (INIA, Spain) presented an approach that allows the study of selective mortality in large cohorts and shows that allele frequency changes occur during the period of high juvenile mortality in *Pinus pinaster* (Robledo-Arnuncio and Unger 2019).

Since trees also pose challenges at the phenotypic level, individual-level data derived from tree rings are currently discussed as sources for descriptors of the tree’s reaction to past environmental stressors. Melania Zacharias (University of Greifswald, Germany) used tree-ring and climatic data to identify years during which trees suffered notably from summer drought, and she was able to identify a set of single-nucleotide polymorphisms (SNPs) shared among trees with similar reaction to drought.

## Conservation strategies

This session addressed the link between genetics, genomics and environment and the conservation of forest genetic resources. An inspiring keynote by Jelena Aleksič (University of Belgrade, Serbia) on the Balkan endemic Omorika spruce (*Picea omorika*) illustrated the utility of genetic knowledge for practical conservation. Based on the patterns of genetic variation, a 10-year program was launched to save both the species and its genetic resources in face of impending dieback. This program, developed by scientists and conservation managers, has irregular funding through projects, a common problem that hinders long-term conservation efforts for both genetic resources and tree species as a whole.

Conservation of forest genetic resources goes hand in hand with their sustainable use. Management practises considering distinct gene pools, levels of genetic diversity and diverse forest reproductive material are all prudent conservation measures especially in rare tree species, as Barbara Fussi (AWG, Germany) stressed in her talk. These measures can increase the richness of forest genetic resources, which in turn leads to a higher adaptive potential to environmental changes (Samuel Belton, National Botanic Gardens, Ireland). Therefore, a network of sites is needed for in situ conservation of genetic resources, complemented by ex situ stands. In Europe, these are called gene conservation units and form the backbone of forest genetic resource conservation (Lefèvre et al. 2013). They are usually managed, but can also be integrated into forest reserves, as in Switzerland (Andreas Rudow, ETHZ, Switzerland) and Serbia (Jelena Aleksič).

Knowledge of patterns of genetic variation is crucial for the efficient use and conservation of forest genetic resources. However, there are still many open questions, e.g. about relevant evolutionary and ecological processes or related to species-specific selection pressures (Julia Geue, Umeå University, Sweden). In this session, we heard some answers: soil variation may play an important role in *Abies religiosa* at the landscape level (Sebastian Arenaz-Jiménez, UNAM, Mexico), and maternal effects can influence early fitness of *Pinus sylvestris*, with better survival strongly correlated with higher seed mass (Azucena Jiménez-Ramírez, INIA, Spain). Tree physiology, habitat and history must also be considered as exemplified for relict oak populations in Germany (Devrim Semizer-Cuming, FVA, Germany).

The session provided valuable insights into relevant research questions that need to be further addressed for effective conservation and use of forest genetic resources. It became clear that this cannot and should not be separated from forest management and the environment, while at the same time it is necessary to ensure a massive commitment of funding to obtain the knowledge needed and apply it on the ground.

## Towards climate-smart forests

This session addressed the contribution of genetic and genomic research towards adjusting forest management to the needs of climate change. Santiago González-Martínez (INRAE, France) opened the session with an enlightening keynote on how genomics can improve predictions of forest tree population response to climate change, exemplifying this in the case of a clonal trial of *Pinus pinaster* (de Miguel et al. 2020). Conditional neutrality and antagonistic pleiotropy among the considered loci pose main challenges (Anderson et al. 2013), while model validation is essential before transferring the results into forest practice. Common gardens will remain one of our best tools for predicting environmental responses of forest trees, as Santiago González-Martínez concluded.

Such trials are useful not only for addressing adaptive variation, but also for elaborating assisted gene flow (AGF) and breeding programs under changing climatic conditions. Based on a GWAS study, Rafael Candido Ribeiro (University of British Columbia, Canada) provided insights into the genetic basis of drought and cold stress hardiness in Douglas fir (*Pseudotsuga menziesii*). These results will provide a basis to support future seed transfer and breeding strategies. Focusing on another North American conifer, red spruce (*Picea rubens*), Susanne Lachmuth (University of Maryland, USA) demonstrated how standardized genomic offsets can be used to inform climate-smart seed transfer. In particular, she illustrated how the presented AGF approach can maximize the representation of within-species adaptive genomic variation, thus increasing forest resilience in the face of climate change.

Provenance and taxonomic identification represent another application field where advances were made possible with the use of genetic and genomic tools. Due to this progress, analysis of thousands of SNP loci is feasible and has greatly improved origin assignment of forest reproductive material (FRM), as shown in the example of the Western Mediterranean *Pinus pinaster* (Sanna Olsson, INIA, Spain). In the case of the European sessile (*Quercus petraea*) and downy oak (*Q. pubescens*), highly informative sets of SNPs enable accurate species identification and assessment of genetic introgression. Oliver Reutimann (WSL and ETH, Switzerland) showed that not only species composition, but also the degree of admixture between these two interfertile species can be predicted using environmental and topographic variables. In the last talk of the session, Lucian Curtu (Transilvania University of Braşov, Romania) reported about genetic differentiation of the two subspecies *Q. robur* ssp. *robur* and ssp. *pedunculiflora*. Results suggest an adaptation of ssp. *pedunculiflora* to xeric site conditions. Both case studies with oaks emphasized the importance of understanding the dynamics of adaptive introgression in the context of climate-smart forestry.

## Conclusions and Outlook

In recent years, continuously growing genomic resources have enabled advances in studying forest ecosystem adaptation. New statistical and computational tools have been developed, while a large number of case studies have improved our understanding of evolutionary dynamics in forest ecosystems. The great variety of contributions in the conference reflected the progress made. As shown by the large interest in this EvolTree conference, the community is growing and genomics is finally a well-established part of forest science. Moreover, genomics is now also prominent among application-oriented studies, whether these focus on conservation or management.

The above-described progress is obvious from previous reports on forest genomic conferences that have taken place in Europe. Around a decade ago, knowledge on candidate genes for adaptive traits made it possible to integrate genomics in studies of evolutionary response of forest trees to climate change (Kremer et al. 2011). In the 2010s, next-generation sequencing (NGS) increasingly gained importance in forest genomic research (Holliday et al. 2017). Reference genomes of several ecologically and economically important forest trees have now been assembled (e.g. Nystedt et al. 2013, Plomion et al. 2018, Mosca et al. 2019). Thanks to these developments, investigating genome-wide adaptation signatures is now feasible in many forest tree species (Fady et al. 2020).

At the same time, as highlighted in this EvolTree conference, genomic signatures of adaptation can be very complex due to the polygenic character of growth-related and functional traits (Csilléry et al. 2018). Moreover, understanding the underlying demography and population structure not only is crucial when assessing adaptation, but also further exacerbates the detection of adaptation signatures in the genome. It is evident that long-term common-garden experiments, covering a wide ecological amplitude of provenances and field sites, remain indispensable in forest genetics. Hence, a better support by funding agencies in forestry research is needed for establishing such trials or maintaining existing ones to ensure the availability of this important tool for the next generation of forest genomics researchers. Their combination with analyses of genomic data appears to be a very promising and powerful way to predict environmental responses of forest trees. New computational and modelling methods have expanded the available toolbox for this purpose (e.g. Fitzpatrick et al. 2021). To this end, advances in phenotyping methods, including dendrochronological and remote sensing approaches, enable a detailed, more efficient and accurate identification of traits under selection (e.g. Depardieu et al. 2021, d'Odorico et al. 2020).

When studying adaptation of forest ecosystems, we have to look beyond a single species and its genome. Symbiosis greatly affects adaptive variation of both forest tree species as hosts and organisms interacting with them, like mycorrhiza, pathogens and parasites. Such interactions may directly or indirectly mediate between trees and their environment with all types of effects from positive to negative. Interactions among individuals of the same species and temporal changes in allele frequencies within a forest stand are topics that certainly deserve more attention. The evolutionary significance of interspecific hybridization was stressed in several talks dealing with various genera of forest trees (firs, pines, oaks). In these genera, considering a species as a fixed evolutionary unit is most likely not purposeful in view of climate-driven dynamics, because adaptive introgression from a sister species might actually facilitate adaptation to a changing environment. Hybridization and introgression will therefore be important topics in future research of climate change genomics.

Genomics has become an integral part of application-oriented studies, as highlighted in the last two conference sessions. Both in conservation and forest management, the application of genomics greatly improves the resolution in identifying population structure, provenances or conservation and management units. Given the current developments, it is expected that genomics will increasingly support schemes for seed selection, assisted gene flow and adaptive forest management in a changing climate.

This inaugural meeting of the new EvolTree conference series attracted a large number of participants and covered a wide range of aspects related to genomics and adaptation of forest ecosystems. It filled an important gap of interaction possibilities caused by the COVID-19 pandemic and we hope that it will stimulate further research in this dynamically developing research area.

## Supplementary Information

ESM 1 (XLSX 13.9 KB)

## Data Availability

All data generated or analyzed during this study are included in this published article (and its supplementary information files).
